# microRNA-622 upregulates cell cycle process by targeting FOLR2 to promote CRC proliferation

**DOI:** 10.1186/s12885-023-11766-6

**Published:** 2024-01-02

**Authors:** Yuehong Chen, Feng Liu, Xinhua Chen, Wenyi Li, Kejun Li, Hailang Cai, Shunyi Wang, Honglei Wang, Ke Xu, Chenxi Zhang, Shengzhi Ye, Yunhao Shen, Tingyu Mou, Shumin Cai, Jianwei Zhou, Jiang Yu

**Affiliations:** 1 Department of General Surgery, Guangdong Provincial Key Laboratory of Precision Medicine for Gastrointestinal Tumor, The First School of Clinical Medicine, Nanfang Hospital, Southern Medical University, Guangzhou, 510515 China; 2 Department of Colorectal and Anal Surgery Guangzhou First People’s Hospital, School of Medicine, South China University of Technology, Guangzhou, 510515 China; 3 Department of Radiology, Nanfang Hospital, Southern Medical University, Guangzhou, 510515 China; 4 Department of Critical Care Medicine, Nanfang Hospital, Southern Medical University, Guangzhou, 510515 China; 5 Department of Critical Care Medicine, The First School of Clinical Medicine, Southern Medical University, Guangzhou, 510515 China; 6 Department of Medical Imaging Center, Nanfang Hospital, Southern Medical University, No. 1838, Guangzhou Avenue North, Guangzhou, 510515 China

**Keywords:** Colorectal cancer, miR-622, FOLR2, Proliferation, Cell cycle

## Abstract

**Background:**

Epigenetic alterations contribute greatly to the development and progression of colorectal cancer, and effect of aberrant miR-622 expression is still controversial. This study aimed to discover miR-622 regulation in CRC proliferation.

**Methods:**

miR-622 expression and prognosis were analyzed in clinical CRC samples from Nanfang Hospital. miR-622 regulation on cell cycle and tumor proliferation was discovered, and FOLR2 was screened as functional target of miR-622 using bioinformatics analysis, which was validated via dual luciferase assay and gain-of-function and loss-of-function experiments both in vitro and in vivo.

**Results:**

miR-622 overexpression in CRC indicated unfavorable prognosis and it regulated cell cycle to promote tumor growth both in vitro and in vivo. FOLR2 is a specific, functional target of miR-622, which negatively correlates with signature genes in cell cycle process to promote CRC proliferation.

**Conclusions:**

miR-622 upregulates cell cycle process by targeting FOLR2 to promote CRC proliferation, proposing a novel mechanism and treatment target in CRC epigenetic regulation of miR-622.

**Supplementary Information:**

The online version contains supplementary material available at 10.1186/s12885-023-11766-6.

## Background

Colorectal cancer (CRC) is one of the prevalent malignant diseases and causes of cancer-related death and health burden globally [1]. It is of great importance to elucidate the mechanisms underlying CRC tumorigenesis and progression, which will further drive discovery of novel diagnostic and prognostic biomarkers, and development of more effective pharmaceuticals.

microRNAs (miR) are 20-to-24 nucleotides small noncoding RNAs, generated by Dicer processed ~ 70-base single-stranded RNA precursor [2–4], exert epigenetic regulation at transcription level by recognizing homologous mRNA sequence, which have been a research hotspot since first discovery in 1993 [5]. Decades of studies have proved that continuous accumulation of genetic and epigenetic alterations contribute to the development and progression of CRC [6, 7], and aberrant miRNA expression in multiple cancer types played important regulator role that is no second to protein-coding genes [8].

Accumulating evidence suggests that miR-622 is differentially expressed and significantly indicates prognosis in several malignancies, such as CRC [9], gastric cancer [10], pancreatic ductal adenocarcinoma [11], lung cancer [12], liver cancer [13], breast cancer [14], serous ovarian carcinoma [15, 16], etc. miR-622 participates in mediating tumor migration and metastasis [14, 17], angiogenesis [13] and chemo-resistance [16]. However, controversy remains in whether it acts as a tumor promoter or suppressor. In our previous work, miR-622 was screened in radiotherapy-resistant CRC cells to be overexpressed and target tumor suppressor RB1 [18].

Herein we report evidence of miR-622 overexpression in CRC, which indicates unfavorable prognosis, and miR-622 promoted CRC proliferation both in vitro and in xenograft tumors through cell cycle pathways. miR-622 specifically targets and downregulates FOLR2, which could in turn attenuated miR-622-induced tumor growth, suggesting a novel mechanism and treatment target in CRC epigenetic regulation.

## Materials and methods

### Clinical specimens

122 Fresh surgical CRC samples and matched normal tissues were collected from Nanfang hospital and histologically confirmed. All patients were treatment naive. Experiment protocols concerning human subjects were consistent with the principles of the Declaration of Helsinki and approved by the Ethics Committee of Nanfang Hospital. All patients given informed consents.

### Mice

Balb/c-nude mice were purchased from the Central Laboratory of Animal Science of Southern Medical University. Mice were bred in specific pathogen-free environment under suitable temperature and light-controlled room with ad libitum food and water. All studies were performed in male mice unless otherwise indicated. Animal related research protocols are consistent with the U.S. Public Health Service Policy on Use of Laboratory Animals, and were approved by the Ethics Committee on Use and Care of Animals of Southern Medical University.

### Cell lines and transient or stable transfection

All human CRC cell lines SW620, RKO, SW480, HCT116, LOVO, LS174.T and HT29 were obtained from ATCC (Manassas, VA, USA). Cells were cultured in RPMI-1640 (Gibco) containing 10% heat-inactivated fetal bovine serum (FBS). Cells were maintained at 37 ℃ in a humidified incubator containing 5% CO_2_. Mycoplasma contamination was tested prior experiment. As previously described [18], for transient transfection, 0.5 µM miR-622 mimics or inhibitor and their negative controls (N.C)were transfected into indicated cells following manufacturer instructions. For stable transfection, lentiviruses containing miR-622 overexpression (LV-miR-622) or inhibition (LV-inhibitor), or FOLR2 overexpression (LV-FOLR2) vectors, and their negative controls (N.C) were transfected into indicated cells using Lipo2000 (Invitrogen). FOLR2 transfection efficacy was tested with immunoblot.

### CCK-8 and colony formation assay

For CCK-8 assay, stable transfected cells were cultivated on 96-well plates (1,000 cells per well) and OD value were detected using CCK-8 (Dojindo, Japan) at 570 nm on Microplate Reader for 6 consecutive days. For colony formation assay, indicated cells were cultivated on 6-well plates (500 cells per well) for 14 days. Plates were washed with PBS, fixed in 70% methanol and stained with 0.1% crystal violet. Colonies containing over 50 cells were counted. All experiments were repeated for three times.

### Immunoblot analysis

Indicated cells were lysed and quantified with BCA Protein Assay Kit (Thermofisher, 23,225). Equal amount of protein lysate was separated by electrophoresis and then transferred to PVDF membrane (IPFL00010, Merck Millipore). After blocking with 5% fully skimmed milk, the PVDF membrane were incubated with the primary antibody anti-FOLR2 (1:1000, 60004-1-Ig, Proteintech) and anti-GAPDH (1:1000, Proteintech). Signal was detected using horseradish peroxidase (HRP)-conjugated secondary antibodies and Super Signal West Femto Chemiluminescent Substrate (34,096, Thermo Fisher Scientific). Images were captured and analyzed using the Image Lab Software (Tanon 5200).

### Immunohistochemistry (IHC) and immunofluorescence (IF) staining

For IHC staining, formalin fixed paraffin-embedded tissues (FFPE) were sectioned, deparaffinated, and incubated with antibody anti-Ki67 (1:500, BD Science, CA, US) or anti-FOLR2 (1:500, #550,609, Bioss) overnight at 4 °C. Non-immune goat serum was used as negative control. Slides were imaged using Olympus BX53 microscope.

### Total RNA extraction and real-time quantitative PCR

Total RNA of cell lines and fresh human tissues were isolated using Trizol reagent (TaKaRa, Dalian, China) following manufacturer’s instruction. cDNA synthesis was performed according to the instruction of PrimeScript™ RT reagent Kit (TaKaRa, Dalian China). qRT-PCR was conducted using SYBR Premix Ex Taq™ II (TaKaRa, Dalian China) and 7500-fast instrument (Applied BioSystems). Data were normalized to snRNA U6 mean Ct value and presented as 2^−ΔΔCt^. Primers used were designed by GeneCopoeia, Inc., Guangzhou, China (Supplementary Table 1).

### Cell cycle detection and flow cytometry

For cell cycle detection, indicated cells were collected and fixed in 70% ethanol overnight before incubated with RNase A and Prodium Iodide (KeyGEN BioTECH) following manufacturer instructions. After washing steps, cytometry was performed on LSRFortessa X-20 (BD Science) and analyzed using FlowJo software (TreeStar).

### Dual luciferase reporter assay

As previously described [18], briefly, DNA sequences containing miR-622 binding site of EPHA7 3’UTR, FOLR2 3’UTR and mutated FOLR2 3’UTR were generated with PCR amplification and subcloned into pGL3-based luciferase reporter plasmid (Promega, US) before cotransfected with control pRL-TK renilla plasmid into cells. Luciferase activity was detected with the Dual Luciferase Reporter Assay Kit (Promega, US) after transfection for 48 h.

### Subcutaneous transplantation

2 × 10^6^ RKO or SW620 cells per mice were subcutaneously transplanted into right back flank of Balb/c-nude mice respectively. Tumor volume were measured and calculated at indicated time points (tumor volume = length× width^2^ × 0.5). When tumor volume reached 2000 mm^3^ or evident signs of ulceration were shown, mice were euthanized with 0.6% amobarbital i.p. before cervical dislocation. Tumors were dissected, measured and photographed at indicated time, then embedded in OCT compound or made into FFPEs for further assessment.

### Statistics and bioinformatics

Statistical parameters are all shown in figure legends. Public datasets used were downloaded from Gene Expression Omnibus (GEO). Survival analysis and optimal cutoff of miR-622 expression was performed using X-tile software [19]. Gene set enrichment analysis (GSEA) was conducted in GSEA software (ver. 4.2.2) [20, 21]. Gene ontology (GO) gene set “c5.go.bp.v7.2” was downloaded from the Molecular Signatures Database (MSigDB). Statistical analysis was performed using nonparametric two-tailed *t* test or two-way ANOVA in GraphPad Prism. Unless otherwise indicated, all experiments were conducted 3 times and data were presented as mean ± SEM (standard error of the mean). *P < 0.05; **P < 0.01; ***P < 0.001.

## Results

### miR-622 overexpression in CRC indicates unfavorable prognosis

**Fig. 1:**
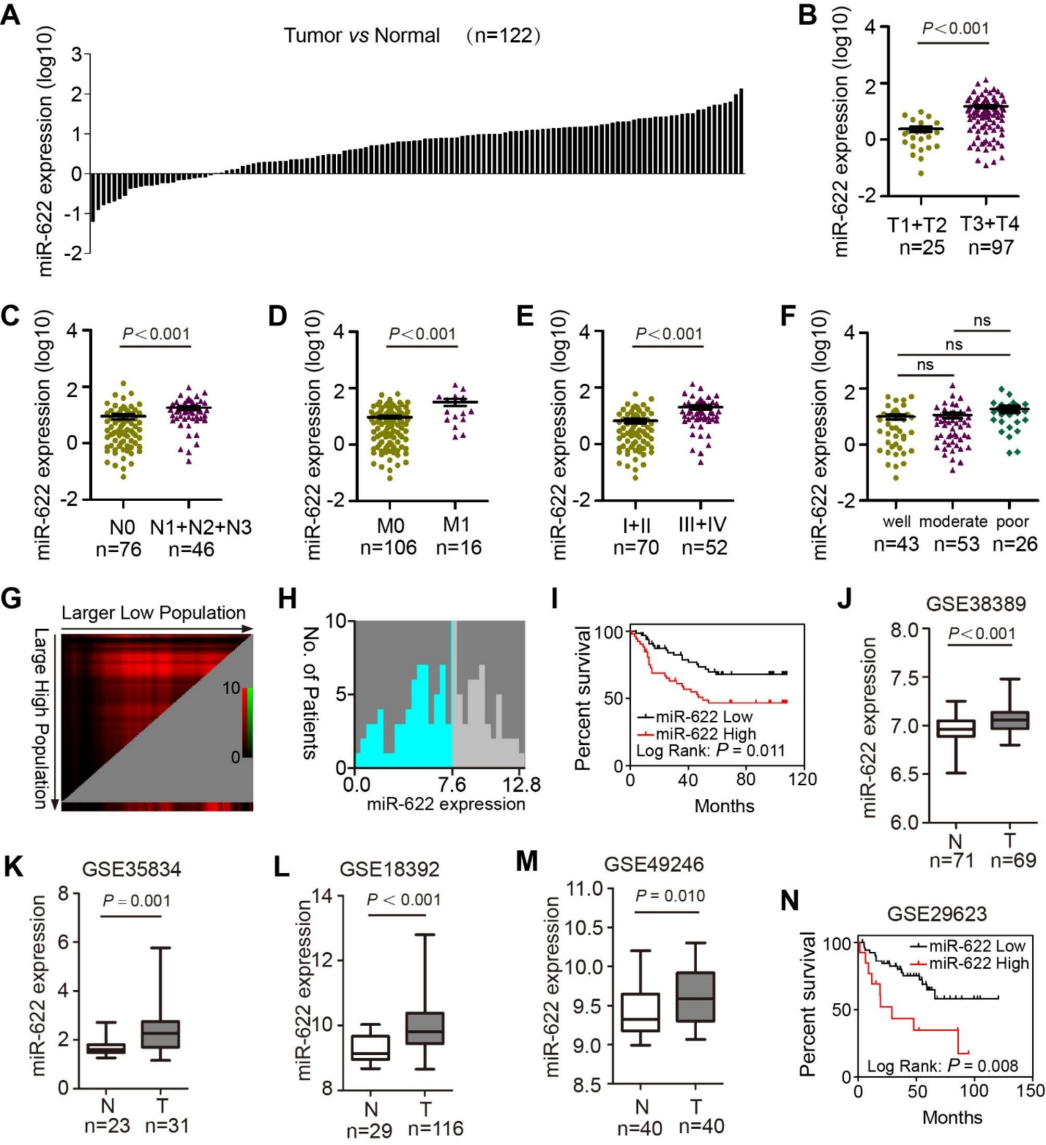
miR-622 is overexpressed and indicates poor prognosis. **(A)** miR-622 relative expression in CRC (paired tumor vs. normal, n = 122, log10 normalized). **(B)** miR-622 relative expression in T stages (T1 + T2 vs. T3 + T4). *P* < 0.001, *t*-test. **(C)** miR-622 relative expression in N stages (N0 vs. N1-3). *P* < 0.001, *t*-test. **(D)** miR-622 relative expression in M stages (M0 vs. M1). *P* < 0.001, *t*-test. **(E)** miR-622 relative expression in different tumor stages (I + II vs. III + IV). *P* < 0.001, *t*-test. **(F)** miR-622 relative expression in differentiation types (well vs. moderate vs. poor). ns, no significance; *t*-test. **(G)**-**(I)** Survival analysis of miR-622 high/low population of patient cohort in 1 A. (G) Larger miR-622 low expressing population shows longer survival time. (H) Optimal cutpoint (7.6) defines high/low population of miR-622 patient cohort in 1 A. (I) miR-622-low population showed higher survival rate. log-rank *P* = 0.011. **(J)-(M)** miR-622 expression in four GSE datasets (GSE38389, GSE35834, GSE18392, GSE49246). N, normal. T, tumor. *t*-test. **(N)** Survival analysis of miR-622 high/low population in GSE29623. log-rank *P* = 0.008

miR-622 expression was detected in 122 paired CRC tumor and normal colon tissues from our center’s biobank, which showed high miR-622 in tumor (Fig. 1A). Subgroup analysis of this cohort showed that miR-622 was higher in patients with greater depth of invasion (T3 + T4; Fig. 1B), one or more lymph node metastases (N1-3; Fig. 1C), distant metastasis (M1; Fig. 1D), or more advanced staging (III+IV; Fig. 1E), but no difference in differentiation types (Fig. 1F). miR-622 expression cutoff found using X-tile indicated that CRC patients with higher miR-622 predicted unfavorable overall survival (log-rank *P* = 0.011; Fig. 1G-I). mir-622 high expression (Fig. 1J-M) and prediction of poor survival (Fig. 1N) in CRC was also validated in several GEO datasets.

### miR-622 regulates cell cycle to promote Tumor proliferation in vitro and in vivo

**Fig. 2:**
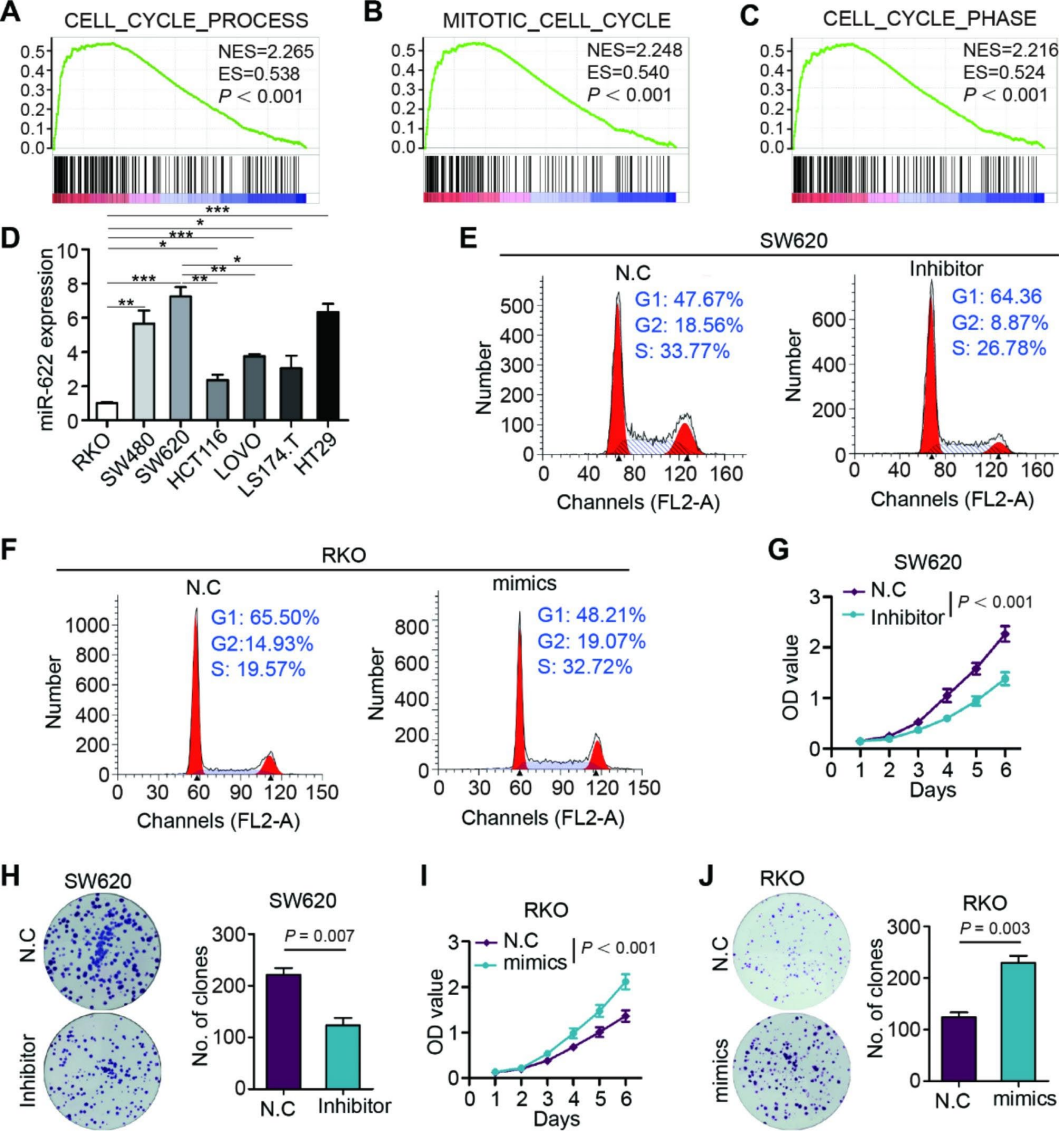
miR-622 regulates cell cycle to promote tumor growth in vitro. **(A)-(C)** GSEA plot of upregulated GO pathways in miR-622 high tumors in GSE29623. **(D)** miR-622 RNA relative expression in human CRC cell lines with RKO group set as reference. Statistical analysis of RKO and SW620 miR-622 expression to other cell lines were shown. * *P* < 0.05, ** *P* < 0.01, *** *P* < 0.001, no significance not shown, *t* test. **(E)** Cell cycle analysis of miR-622 inhibited SW620 using flow cytometry. **(F)** Cell cycle analysis of miR-622 mimic-transfected RKO using flow cytometry. **(G)** CCK-8 assay of miR-622 inhibited SW620. *P* < 0.001, two-way ANOVA. **(H)** Colony formation assay of miR-622 inhibited SW620. *P* = 0.007, *t* test. **(I)** CCK-8 assay of miR-622 mimic-transfected RKO. *P* < 0.001, two-way ANOVA. **(J)** Colony formation assay of miR-622 mimic-transfected RKO. *P* = 0.003, *t* test. All experiments were repeated for three times

Gene set enrichment analysis suggested that pathways enriched in miR-622 high group related to cell cycle, including “cell cycle process”, “mitotic cell cycle” and “cell cycle phase” (Fig. 2A-C), which enlightened us to investigate the mechanism underlying miR-622 unfavorable overexpression. Among seven CRC cell lines, SW620 was ranked the highest and RKO was the lowest in miR-622 expression (Fig. 2D). SW620 cell proportion was increased in G1 phase and decreased in S-G2 phases when treated with miR-622 inhibitor (Fig. 2E), with downregulated expression of cell cycle sub-phase markers, such as CCND1, CCNE1, CDK1 and CDK6 (Supplementary Fig. 1A). miR-622 mimics significantly increased RKO cell proportion in S-G2 phases (Fig. 2F), with increased expression of CCNA2, CCNB1, CCNB2, CDK4, CDK6, PCNA and SPK2 (Supplementary Fig. 1B-C). miR-622 inhibitor attenuated SW620 cell growth in CCK-8 assay (Fig. 2G), and reduced colony formation (Fig. 2H). On the contrary, miR-622 mimics significantly increased RKO cell growth (Fig. 2I), increased colony formation (Fig. 2J), while miR-622 mimics significantly increased.

**Fig. 3:**
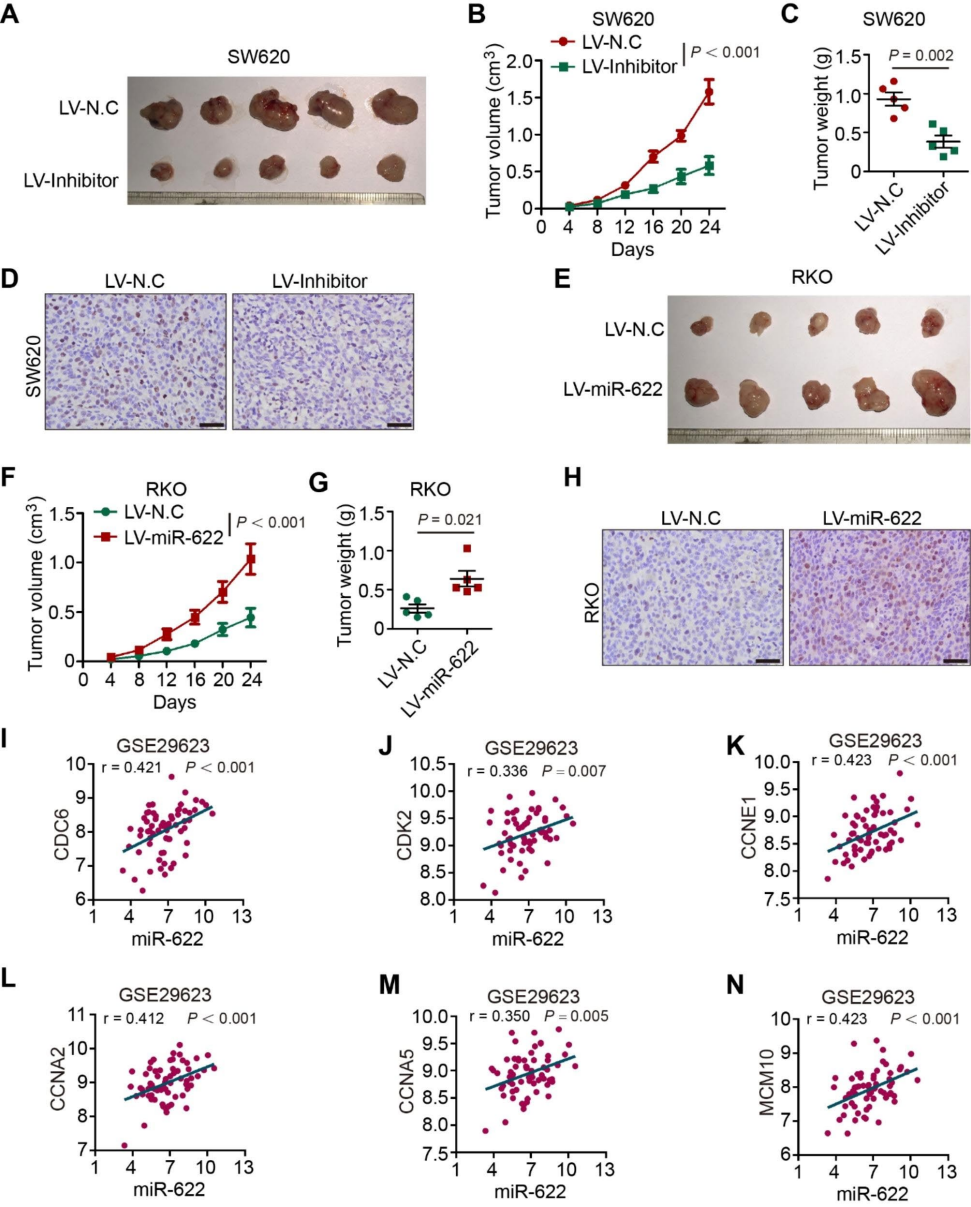
miR-622 promotes tumor growth in vivo. **(A)** Subcutaneous xenograft of lentivirus transfected miR-622 inhibited SW620 (LV-inhibitor) and control group (LV-N.C) in Balb/c-nude mice (*n* = 5). **(B)** Growth curve of subcutaneous xenograft of SW620 (LV-inhibitor) and control group. *P* < 0.001, two-way ANOVA. **(C)** Tumor weight (*g*) comparison between subcutaneous xenograft of SW620 (LV-inhibitor) and control group. *P* = 0.002, *t*-test. **(D)** Representative images of Ki-67 staining in subcutaneous xenograft of SW620 (LV-inhibitor) and control group (LV-N.C). **(E)** Subcutaneous xenograft of lentivirus transfected miR-622 overexpressed RKO (LV-miR-622) and control group (LV-N.C) in Balb/c-nude mice (*n* = 5). **(F)** Growth curve of subcutaneous xenograft of RKO (LV-miR-622) and control group. *P* < 0.001, two-way ANOVA. **(G)** Tumor weight (*g*) comparison between subcutaneous xenograft of RKO (LV-miR-622) and control group. *P = 0.021, *t*-test. **(H)** Representative images of Ki-67 staining in subcutaneous xenograft of RKO (LV-miR-622) and control group (LV-N.C). **(I)**-**(N)** Correlation analysis between miR-622 and CDC6, CDK2, CCNE1, CCNA2, CCNA5 in GSE29623 dataset. Pearson’s r

SW620 transfected with lentivirus-miR-622-inhibitor or –negative control (N.C) was injected subcutaneously. miR-622 inhibited group formed smaller tumors, showed a slower growth curve and smaller tumor weight (Fig. 3A-C). As indicator of cell proliferation, Ki-67 staining showed less proliferating tumor cells in miR-622 inhibited group (Fig. 3D). Subcutaneous tumor model of RKO cells transfected with miR-622 overexpression (OE) and –N.C lentivirus was also established. Overexpressed group formed larger tumors, showed a faster growth curve and bigger tumor weight (Fig. 3E-G). Ki-67 positive cells were detected more in miR-622 overexpressed group (Fig. 3H). Analysis of dataset GSE29623, an mRNA and microRNA profile in colon cancer, supported that miR-622 positively correlated with signature genes in cell cycle pathway (Fig. 3I-N), such as CDC6 (r = 0.421), CDK2 (r = 0.336), CCNE1 (r = 0.423), CCNA2 (r = 0.412), CCNA5 (r = 0.350), and MCM10 (r = 0.423). These results indicated pro-tumor proliferation of miR-622 through regulating cell cycle process both in vitro and in vivo.

### FOLR2 is a functional target of miR-622 to promote CRC proliferation

**Fig. 4:**
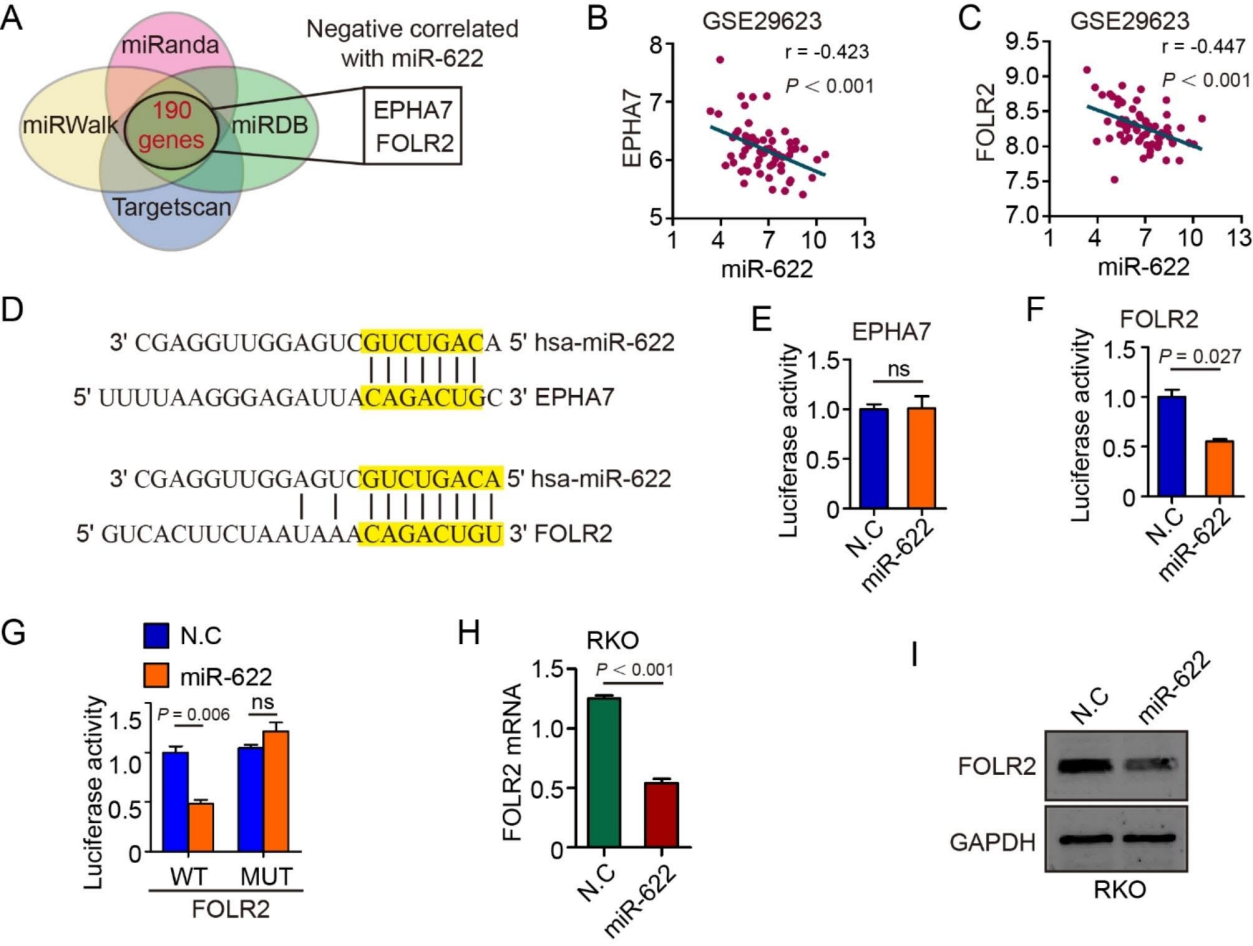
FOLR2 is a functional target of miR-622. **(A)** Venn plot of EPHA7 and FOLR2 among 190 genes negatively correlated with miR-622 predicted in four prediction algorithms (miRanda, TargetScan, miRWalk, miRDB). **(B)** Negative correlation between EPHA7 and miR-622 (r = -0.423, P < 0.001). **(C)** Negative correlation between FOLR2 and miR-622 (r = -0.447, P < 0.001). **(D)** Schematics of highlighted putative miR-622-binding sequence within the 3’-UTR of EPHA7 or FOLR2 mRNA. **(E)-(G)** Relative luciferase activity measured after co-transfection of miR-622 encoding plasmid and reporter plasmid containing either the wild-type (WT) sequence of the EPHA7 or FOLR2 3’-UTR, or mutated FOLR2 3’-UTR (MUT). ns, no significance. *t*-test. **(H)** FOLR2 mRNA level in miR-622-overexpressed RKO and control group (RKO-N.C) using qRT-PCR. *P* < 0.001. **(I)** Immunoblot of FOLR2 and GAPDH in miR-622-overexpressed RKO and control group (RKO-N.C). GAPDH was used as internal reference

To explore molecular mechanism by which miR-622 promotes CRC proliferation, four prediction algorithms (miRanda, TargetScan, miRWalk, miRDB) were used to analyze target genes of miR-622. EPHA7 and FOLR2 ranked high among 190 predicted genes, negatively correlated with miR-622 in the intersection of four algorithms (Fig. 4A). In GSE29623 dataset, negative correlation was confirmed between miR-622 and EPHA7 (Fig. 4B; r = -0.423), miR-622 and FOLR2 (Fig. 4C; r = -0.447). Luciferase reporter assay was then performed to determine whether EPHA7 or FOLR2 was a direct target of miR-622. The targeted 3ʹ-untranslated region (UTR) sequences of EPHA7 and FOLR2 were cloned into luciferase reporter vector respectively (Fig. 4D). Transient transfection of the two vectors accompanied with miR-622-mimics into 293T cells led to a significant decrease in luciferase activity of FOLR2 3ʹ-UTR vector group, but not the one with EPHA7 3ʹ-UTR (Fig. 4E-F, P = 0.027). We then constructed luciferase reporter vector containing mutated sites of FOLR2 3ʹ-UTR and co-transfected with miR-622-mimics into 293T cells, which abolished the miR-622-induced decrease in luciferase activity (Fig. 4G). FOLR2 mRNA and protein level were decrease in miR-622 OE RKO cells (Fig. 4H&I), which confirmed that FOLR2 is a target gene of miR-622. Together, these results indicated that miR-622 targets and downregulates FOLR2 mRNA in CRC.

**Fig. 5:**
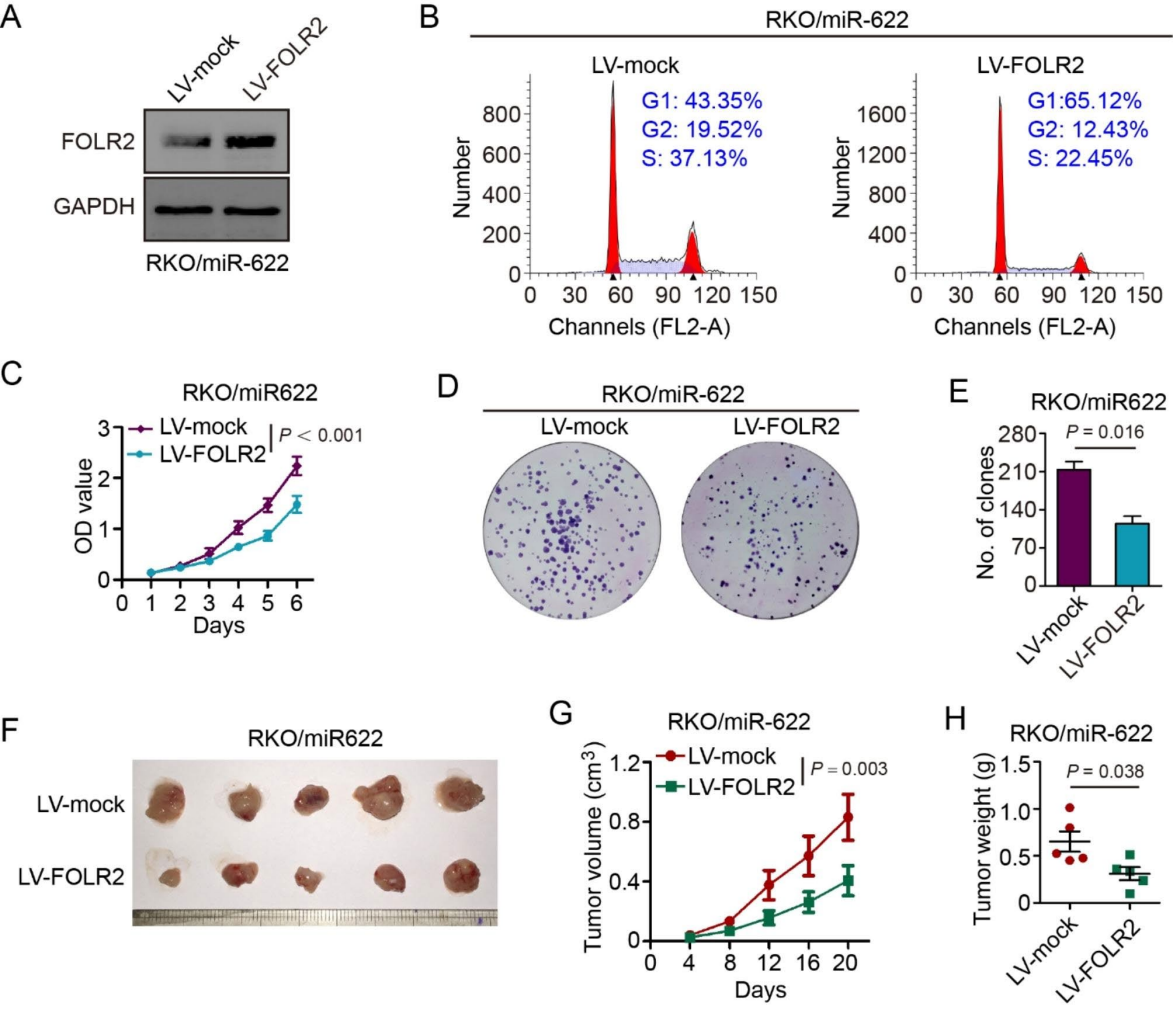
FOLR2 reverses miR-622-induced CRC proliferation. **(A)** Immunoblot of FOLR2 overexpression by lentivirus transfection (LV-FOLR2/-mock) in miR-622 mimic-transfected RKO cells (RKO/miR-622). GAPDH was used as internal reference. **(B)** Cell cycle proportion of RKO/miR-622 (LV-FOLR2) and control group (LV-mock) using flow cytometry. **(C)** CCK-8 assay of RKO/miR-622 (LV-FOLR2) and control group. *P* < 0.001, two-way ANOVA. **(D)-(E)** Colony formation assay of RKO/miR-622 (LV-FOLR2) and control group (LV-mock). *P* = 0.016, *t* test. **(F)** Subcutaneous xenograft of RKO/miR-622 (LV-FOLR2) and control group (LV-mock). **(G)** Growth curve of subcutaneous xenograft in RKO/miR-622 (LV-FOLR2) and control group (LV-mock). *P* = 0.003, two-way ANOVA. **(H)** Tumor weight (*g*) comparison between subcutaneous xenograft in RKO/miR-622 (LV-FOLR2) and control group (LV-mock). *P* = 0.038, *t*-test

To validate whether miR-622 effect on CRC proliferation was indeed achieved through targeting FOLR2 function in CRC, we transfected FOLR2 OE lentivirus (LV-FOLR2) into miR-622 OE RKO cells (Fig. 5A). Overexpressed FOLR2 reduced S-G2 phases cell proportion that elevated by miR-622 (Fig. 5B). CCK-8 assay and colony formation also indicated that overexpression of FOLR2 could partially eliminate CRC proliferation induced by miR-622 (Fig. 5C-E). RKO/miR-622 cells transfected with LV-FOLR2 or –mock were injected to nude mice to form subcutaneous xenograft, and FOLR2 overexpressed group grew smaller and slower compared with control group (Fig. 5F-H). Altogether, these results indicated that miR-622 targets and downregulates FOLR2 mRNA to promote CRC proliferation.

### FOLR2 negatively correlates with signature genes in cell cycle process

**Fig. 6:**
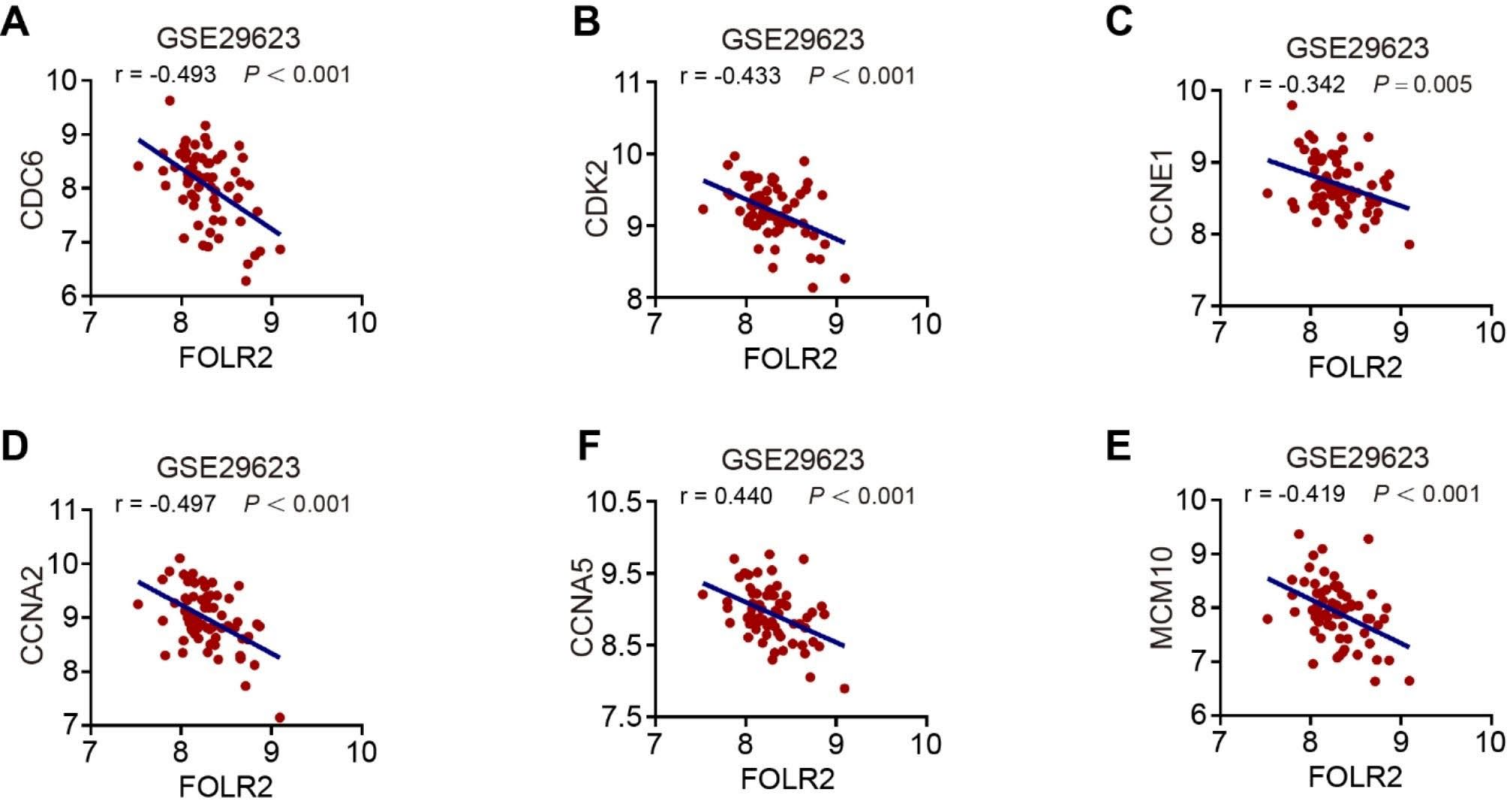
FOLR2 negatively correlates with cell cycle signature genes. **(A)**-**(E)** Correlation analysis between FOLR2 and CDC6, CDK2, CCNE1, CCNA2, CCNA5 in GSE29623 dataset. Pearson’s r

Since miR-622 upregulated cell cycle to promote tumor growth, we further verified correlation between FOLR2 and cell cycle process. FOLR2 was negatively correlated with signature genes in cell cycle pathway (Fig. 6A-E), and correlation coefficient was CDC6 (r = -0.493), CDK2 (r = -0.433), CCNE1 (r = -0.342), CCNA2 (r = -0.497), CCNA5 (r = -0.440), and MCM10 (r = -0.419), respectively. These results indicated that FOLR2 had negative correlation with signature genes in cell cycle process.

## Discussion

Cancer pathogenesis greatly attributes to miRNA dysregulation, whether one may originally serve as tumor suppressor or promoter in such context [8]. In this study, miR-622 was differentially upregulated in both CRC human samples and cell lines, correlated with advanced TNM stages and unfavorable survival prognosis in clinical data. Aberrant miR-622 expression remarkably increased CRC proliferation both in vivo and in vitro through affecting cell cycle process. Four databases predicted significant negative correlation between miR-622 and FOLR2, and dual luciferase reporter assay verified that miR-622 targeted FOLR2 mRNA 3’UTR and down-regulated FOLR2, a downregulated protein in CRC whose overexpression abolished miR-622 pro-tumor effect and showed countering effect to signature genes in cell cycle process.

As a CRC-associated miRNA specifically upregulated in microsatellite stable tumor [9, 22], controversies have remained in the role that miR-622 exerts in tumorigenesis in different cancers since its discovery and exploration by many predecessors. Our findings provided evidence that miR-622 was a tumor promotor in CRC. However, miR-622 often shows contradicting effects through targeting different genes in different tumors. For example, Choi et al. revealed that miR-622 induced resistance to PARPis and platinum in BRCA1-mutant ovarian cancer by targeting the Ku complex and restoring HR-mediated DSB repair [16]; Wang et al. reported that miR-622 targeted DYRK2 to promote the migration and invasion of colorectal cancer [17]; while Liu et al. found that EZH2 inhibited the targeted regulation of miR-622 to upregulate CXCR4 and promote HCC tumorigenesis [13]. Moreover, several studies have reported that circRNA or lncRNA could sponge miR-622 and suppress its original function in different malignacies [12, 23], which prompted us to further explore the complexity of miR-622 regulating mechanism in CRC.

FOLR family has a high affinity for folate and several reductive folate derivatives. FOLR2 gene encodes a protein originally identified as a membrane receptor that mediates the delivery of 5-methyl tetra hydro folate (MTHF) into the cell [24]. In recent years, studies have mainly focused on FOLR2^+^ macrophages in the tumor microenvironment and their role in immunotherapy [25–27]. Xu et al. reported that siRNA-silenced FOLR2 gene could inhibit the phosphorylation of AKT, mTOR and S6K1 to inhibit cell proliferation and increase apoptosis [28], however this work is limited in vitro and lacking more direct evidence, which still in a way indicated FOLR2 function in tumor cells. On the other hand, Mayanil’s laboratory reports suggested that FOLR1 was capable of translocating from cytoplasm into the nucleus to function as a transcription factor that directly regulated gene expression [29–31]. Using a candidate gene approach, they revealed that FOLR1 cis-regulated pluripotency signature genes, upregulated Oct4, Sox2 and Klf4, downregulated miR-138 (targeting Oct4) and miRlet-7 (targeting Trim71) by binding to their enhancer/promoter regions, to help pre-migratory neural crest cells maintain their multipotent phenotype and their proliferation potential prior to differentiation; or lead to phenotypic switching of differentiated glial cells to dedifferentiated cells [29–31]. Since FOLR2 is an important homolog of FOLR1, we hypothesized that it might also function similarly as a transcription factor. Based on our findings, we could further assume that FOLR2 plays an opposite role to FOLR1 in balancing cell proliferation, just like the competitive inhibition between homologous transcription factor IRF1 and IRF2 [32, 33]. However, these theories require further experimental evidence.

## Conclusions

Our study confirms that miR-622 overexpression indicates unfavorable prognosis in CRC, promotes CRC proliferation through cell cycle pathway activation by targeting and downregulating FOLR2. FOLR2 overexpression reduced cell proliferation elevated by miR-622, suggesting a novel mechanism and treatment target in CRC epigenetic regulation of miR-622.

### Electronic supplementary material

Below is the link to the electronic supplementary material.

Supplementary Material 1

## Data Availability

The datasets supporting the conclusions of this article, GSE38389 [34], GSE35834 [35, 36], GSE18392 [37], GSE49246 [38], GSE29623 [39] are available in Gene Expression Omnibus (GEO) database (https://www.ncbi.nlm.nih.gov/geo/).

## Abbreviations

CRC: Colorectal cancer
miR-622: micro ribonucleic acid-622
FOLR2: Folate Receptor Beta 2
RB1: Retinoblastoma 1
ATCC: American type culture collection
DMEM: Dulbecco’s modified Eagle’s medium
FBS: Fetal bovine serum
LV: Lentivirus
CCK-8: Cell counting kit 8
PBS: Phosphate buffer saline
PVDF: Polyvinylidene fluoride
GAPDH: Glyceraldehyde-phosphate dehydrogenase
HRP: Horseradish peroxidase
EPHA7: Ephrin Receptor A7
OCT: Opti-mum Cutting Temperature Compound
FFPE: Formalin fixed paraffin-embedded
GSEA: Gene set enrichment analysis
GO: Gene ontology
ANOVA: Analysis of variance
SEM: Standard error of the mean
GEO: Gene Expression Ominbus
UTR: Untranslated region
OE: Overexpression
PARPi: Poly-ADP-ribose polymerase
DSB: DNA double strand breakage
HCC: Hepatocellular carcinoma
