# Supplementary material for: microRNA-622 upregulates cell cycle process by targeting FOLR2 to promote CRC proliferation
Source: BMC Cancer. 2024 Jan 2;24:26. doi: 10.1186/s12885-023-11766-6 (PMC10763126; doi:10.1186/s12885-023-11766-6)
Supplement: Supplementary file 1 — Supplementary Material 1 [file 12885_2023_11766_MOESM1_ESM.pdf]

Supplementary information

Title page

Title

microRNA-622 upregulates cell cycle process by targeting FOLR2 to promote CRC proliferation

Authors

Yuehong Chen†, Feng Liu†, Xinhua Chen†, Wenyi Li, Kejun Li, Hailang Cai, Shunyi Wang, Honglei Wang, Ke Xu, Chenxi Zhang, Shengzhi Ye, Yunhao Shen, Tingyu Mou, Shumin Cai[*], Jianwei Zhou[*], Jiang Yu[*].

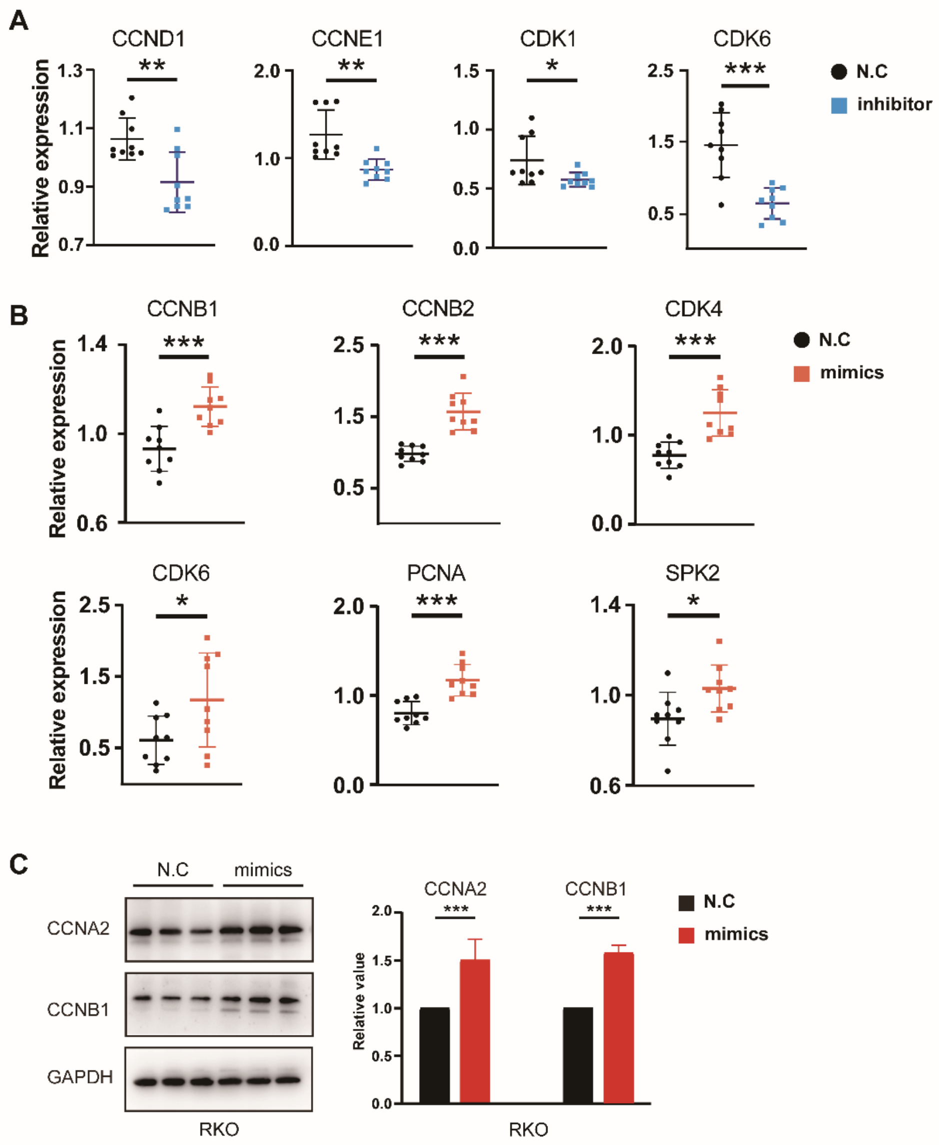

Supplementary Figure 1. (A) CCND1, CCNE1, CDK1 and CDK6 relative expression in miR-622 inhibitor/negative control vectors (N.C) transfected SW620. HPRT expression set as reference. * $P < 0.05$, ** $P < 0.01$, *** $P < 0.001$, *t* test. **(B)** CCNB1, CCNB2, CDK4, CDK6, PCNA and SPK2 relative expression in miR-622 mimics/negative control vectors (N.C) transfected RKO. HPRT expression set as reference. * $P < 0.05$, ** $P < 0.01$, *** $P < 0.001$, *t* test. All experiments were repeated for three times. (**C)** Immunoblots of CCNA2 and CCNB1 in miR-622 mimics/negative

control vectors (N.C) transfected RKO. GAPDH set as internal reference. Statistical analysis of immunoblot was calculated using imageJ and normalized to N.C group respectively. * $P < 0.05$, ** $P < 0.01$, *** $P < 0.001$, *t* test.

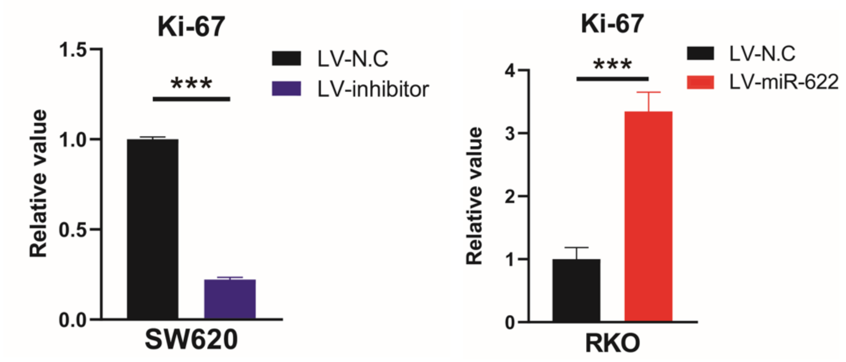

Supplementary Figure 2. Statistical analysis of immunoblot of Fig. 3D & 3E. Ki-67^{+} cells were calculated using imageJ "cell counter" and normalized to N.C group. *** $P < 0.001$, t test. Repeated for three times.

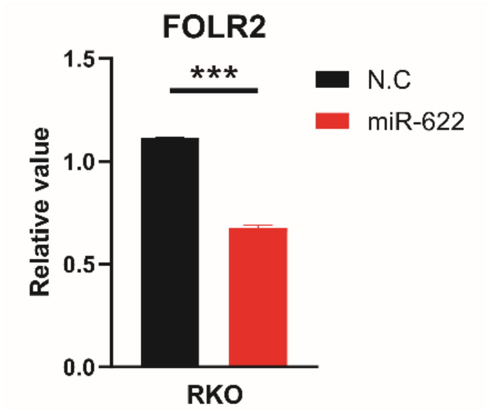

Supplementary Figure 3. Statistical analysis of immunoblot of Fig. 4I. Calculated using imageJ and normalized to GAPDH respectively. *** $P < 0.001$, t test. Repeated for three times.

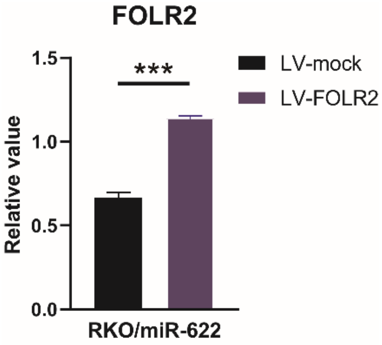

Supplementary Figure 4. Statistical analysis of immunoblot of Fig. 5A. Calculated using imageJ and normalized to GAPDH respectively. *** $P < 0.001$, t test. Repeated for three times.

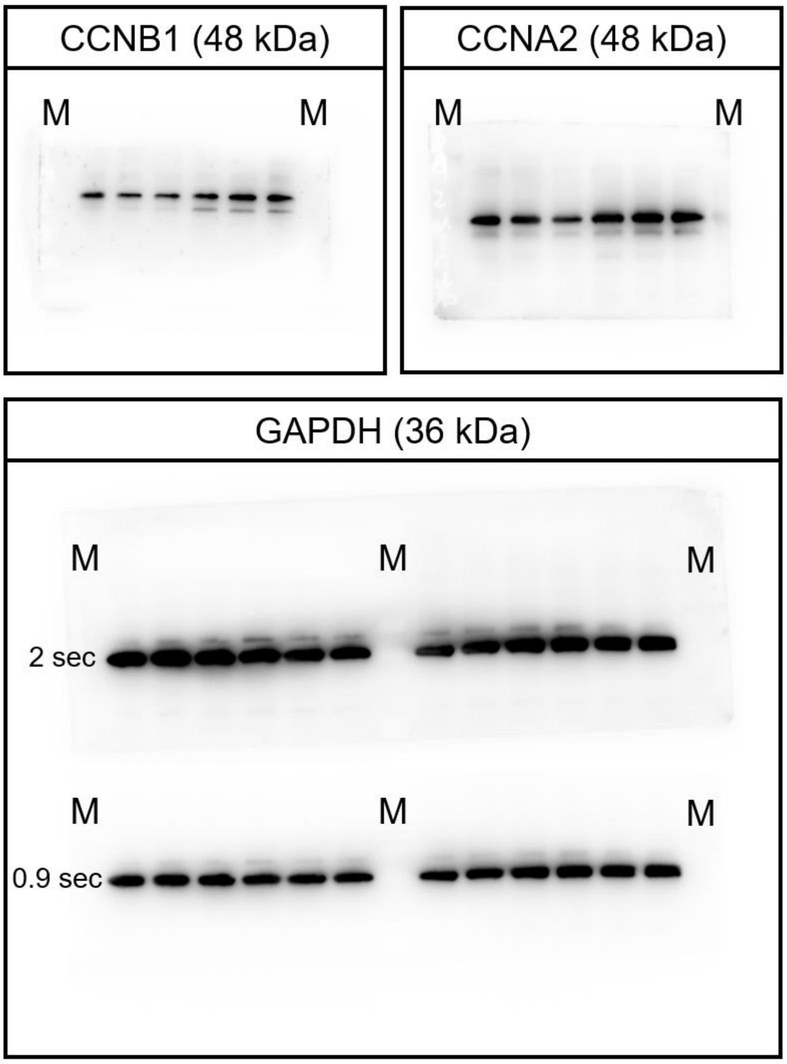

Supplementary Figure 5. Original blot of Supplementary Figure 1C (M, marker lane).

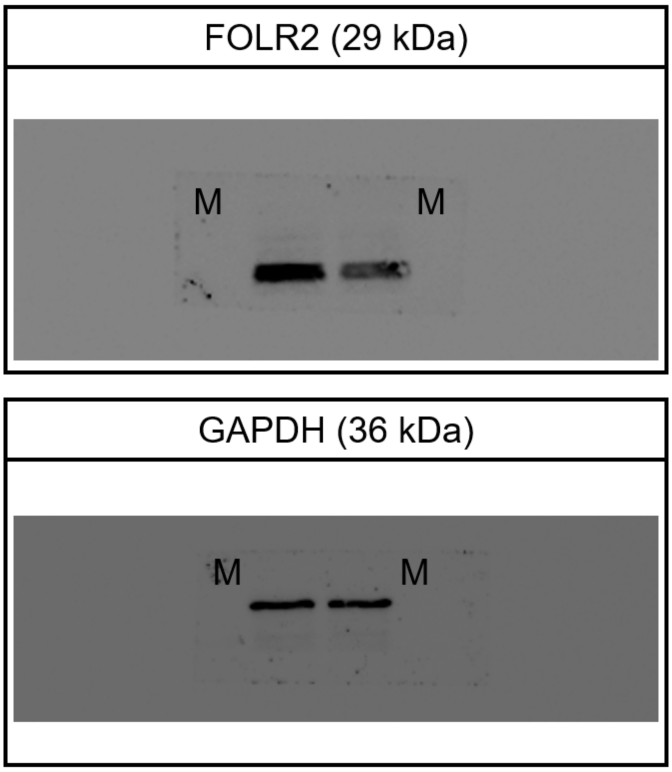

Supplementary Figure 6. Original blot of Figure 4I (M, marker lane).

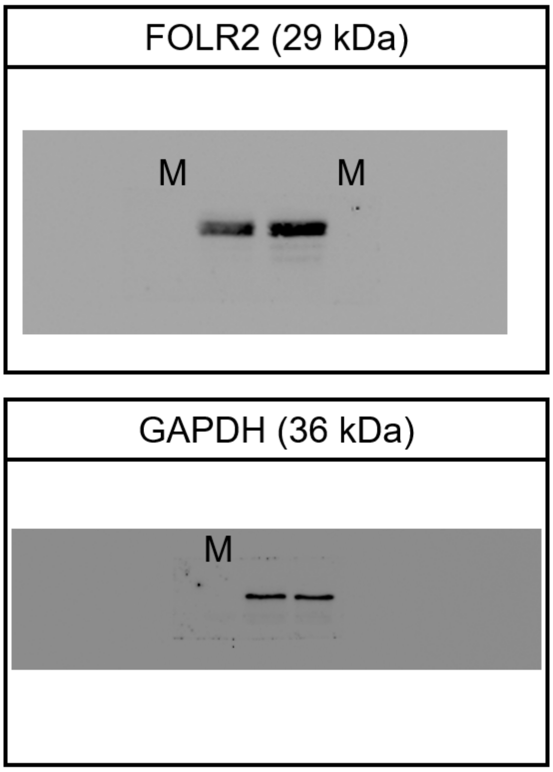

Supplementary Figure 7. Original blot of Figure 5A (M, marker lane).

Supplementary Table 1.

Primers used in this manuscript

	Forward	Reverse
CCNB1	AATAAGGCGAAGATCAACATGGC	TTTGTTACCAATGTCCCCAAGAG
CCNB2	CCGACGGTGTCCAGTGATTT	TGTTGTTTTGGTGGGTTGAACT
CCND1	GCTGCGAAGTGGAAACCATC	CCTCCTTCTGCACACATTTGAA
CCNE1	AAGGAGCGGGACACCATGA	ACGGTCACGTTTGCCTTCC
CDK1	AAACTACAGGTCAAGTGGTAGCC	TCCTGCATAAGCACATCCTGA
CDK4	ATGGCTACCTCTCGATATGAGC	CATTGGGGACTCTCACACTCT
CDK6	GCTGACCAGCAGTACGAATG	GCACACATCAAACAACCTGACC
PCNA	CCTGCTGGGATATTAGCTCCA	CAGCGGTAGGTGTCGAAGC
SKP2	ATGCCCCAATCTTGTCCATCT	CACCGACTGAGTGATAGGTGT
